# Mechanisms for ·${\text{O}}_{2}^{-}$ and ·OH Production on Flowerlike BiVO_4_ Photocatalysis Based on Electron Spin Resonance

**DOI:** 10.3389/fchem.2018.00064

**Published:** 2018-03-26

**Authors:** Xuan Xu, Yaofang Sun, Zihong Fan, Deqiang Zhao, Shimin Xiong, Bingyao Zhang, Shiyu Zhou, Guotao Liu

**Affiliations:** ^1^Key Laboratory of Three Gorges Reservoir Region's Eco-Environment, Ministry of Education, Chongqing University, Chongqing, China; ^2^National Centre for International Research of Low-Carbon and Green Buildings, Chongqing University, Chongqing, China; ^3^College of Environmental and Resources, Chongqing Technology and Business University, Chongqing, China

**Keywords:** electron spin resonance (ESR), degradation mechanism, bismuth vanadate (BiVO_4_), superoxide radical (·O^−^_2_), hydroxyl radical (·OH)

## Abstract

Many studies have focused on the use of BiVO_4_ as a photocatalyst, but few have investigated the production of free radicals during the photocatalytic process. Following synthesis of flowerlike BiVO_4_ and characterization by X-ray diffraction (XRD), Raman spectroscopy, Scanning electron microscopy (SEM) Scanning electron microscopy (EDX), UV-Vis and XPS, we successfully prepared BiVO_4_. Then we used electron spin resonance (ESR) to determine the production and degradation of individual active free radicals, including the superoxide radical (·${\text{O}}_{2}^{-}$) and the hydroxyl radical (·OH). In the first experiment, we used ESR to detect the signals of free radicals (·${\text{O}}_{2}^{-}$ and ·OH) under varying oxygen conditions. The results shown that in addition to production by ·${\text{O}}_{2}^{-}$, ·OH could also be produced by oxidation of h^+^ to OH^−^. In the next experiment, we detected ·OH under varying pH to identify the result of the first experiment, and found that signal intensities increased with increasing pH, indicating the mechanism for ·OH production. Finally, we conducted a trapping experiment to examine free radical degradation mechanisms. We identified ·OH and h^+^ as the main active free radicals and showed the complete production about ·OH. These results improve current knowledge of free radical production mechanisms, which can be used to enhance the photocatalytic performance of BiVO_4_.

## Introduction

The development of modern society and global economy has led to a series of environmental problems. The public's awareness of environmental issues has gradually increased with the growing realization that the environment requires continuous protection in order to sustain life (Zhang et al., 2010). Related to this area of thought is the utilization of solar energy. The development of photocatalytic semiconductors for organic pollutant degradation in wastewater has become a challenging research topic (Zhang et al., 2015). Many studies are now focusing on the photocatalytic decomposition of wastewater and degradation of organic pollutants under visible light irradiation (Long et al., 2006). Since the discovery of photo-induced decomposition of water by TiO_2_ electrodes (Inoue et al., 1979), semiconductor-based photocatalysis has attracted extensive interest and the properties of several photocatalytic materials, such as TiO_2_ and ZnO, have been investigated. The development of visible-light-driven photocatalysts involves two strategies (Long et al., 2006): modification of TiO_2_ and exploitation of novel semiconductor materials. Many studies have reported that TiO_2_ is a promising material for Surface Enhanced Raman Scattering (SERS) owning to its high refractive index, versatility in terms of surface functionalization, and synergistic coupling with plasmonic nanoparticles (Xu et al., 2017). In addition, TiO_2_ has many advantages that are beneficial to photocatalytic reactions, such as extraordinary chemical stability, low cost, and small environmental impact (Inoue et al., 1979). However, it also has some limitations, such as a low quantum efficiency and wide band-gap (3.2 eV); therefore, TiO_2_-based photocatalytic reactions can only occur under ultraviolet irradiation (Zhang et al., 1998; Ren et al., 2012; Wang et al., 2014). Bismuth vanadate (BiVO_4_), a promising photocatalyst, has received considerable attention because of its high absorption of visible light (VL), and VL accounts for over 50% of total sunlight (Xu et al., 2016). Due to its non-toxicity, environmentally friendly BiVO_4_-based paints have recently replaced paints containing toxic pigments, such as lead chromate (PbCrO_4_) and cadmium sulfide (CdS) (Nalbandian et al., 2015). As a new VL-active photocatalyst, BiVO_4_ occurs in three main crystal forms: monoclinic scheelite, tetragonal zircon, and tetragonal scheelite structures. Monoclinic scheelite and tetragonal scheelite possess similar scheelite crystalline structures (Shan et al., 2014). Compared with the other two phases, the monoclinic scheelite phase of BiVO_4_ exhibits much higher photocatalytic activity under visible light, as illustrated by extensive photocatalytic research (Xu et al., 2016). With the rise of BiVO_4_ applications, various methods for the synthesis of BiVO_4_ photocatalysts have been proposed (Zhao et al., 2016). In addition, many researchers have focused on modifying BiVO_4_ to inhibit the recombination of electron carriers and improve its photocatalytic absorption efficiency (Kohtani et al., 2005; Xu et al., 2008; Wang et al., 2015; Zhang et al., 2016; Zhao et al., 2016).

Though many studies have focused on the modification of BiVO_4_ and its photocatalytic properties, there are few published investigations on its photocatalytic mechanism. According to published literatures (Hashimoto et al., 2005; Lam et al., 2014), the main active free radicals that take part in the photocatalytic system include the hydroxyl radical (·OH), the superoxide radical (·${\text{O}}_{2}^{-}$), and the hole (h^+^). Among these active free radicals, ·OH is mainly generated by oxidation of h^+^ to water or via a series of reactions involving ·${\text{O}}_{2}^{-}$. The main activate free radicals that affecting the degradation of photocatalytic materials in different photocatalytic systems vary, although they typically include one of the three above-mentioned radicals. Previous research has focused on the direct effects that photocatalytic materials exert on pollutant degradation, and reaction mechanisms typically are based on trapping experiments (Xu et al., 2016). However, there is limited research on identification of the main active free radicals using various methods, and the mechanisms of ·OH and ·${\text{O}}_{2}^{-}$ generation are unclear (Ge et al., 2012b). Some studies on BiVO_4_ have reported the reduction of oxygen by electrons as a mechanism for the generation of ·${\text{O}}_{2}^{-}$, however no experiments have been conducted to confirm this conclusion. Furthermore, some studies have reported conflicting conclusions on free radical reaction mechanisms, indicating that ·OH can be generated by ·${\text{O}}_{2}^{-}$ but not by h^+^ (Cheng et al., 2012). Thus, it is unclear whether ·OH can be generated by h^+^, and further research is necessary to clarify the mechanisms of free radical reactions.

Spin trapping in conjunction with electron spin resonance (ESR) has been widely used for the indirect detection of short-lived radicals (Ming et al., 2014). Spin trapping compounds are used to convert short-lived radicals into relatively longer-lived radical products, spin adducts, which can be easily detected by ESR (Castellanos et al., 2001; Kubo et al., 2012). This is a very intuitive method to detect the presence of unpaired electrons, such as free radicals, transition metals, electron holes, etc.

The aim of this study was to clarify the recent controversy regarding the formation mechanism of ·OH, particularly whether it can be generated by ·${\text{O}}_{2}^{-}$ or h^+^. In addition to the synthesis and characterization of BiVO_4_, we investigated its photocatalytic degradation mechanism with regard to the formation of ·OH. We used ESR to determine individual active free radicals, including ·${\text{O}}_{2}^{-}$ and ·OH. According to the results, oxygen plays multiple roles in this mechanism and primarily determines the pathway by which these two active free radicals are generated. We further investigated the main radicals participating in the photocatalytic process by changing the pH of the reaction system. Finally, a trapping experiment was conducted and the photocatalytic mechanism of BiVO_4_ was discussed in detail.

## Experimental methods

### Reagents, preparation, and characterization of materials

#### Reagents

Pure chemicals of analytical grade were used without further purification. Sodium hydroxide (NaOH), ammonium metavanadate (NH_4_VO_3_), and silver nitrate (AgNO_3_) were purchased from the Chongqing Chuandong Chemical Company. Bismuth nitrate (Bi (NO_3_)_3_·5H_2_O) and nitric acid (HNO_3_) were obtained from the Chengdu Area of the Industrial Development Zone (Xinde Mulan). Dimethyl pyridine N-oxide (DMPO), absolute ethyl alcohol (C_2_H_5_OH), and isopropanol (IPA) were acquired from the Industrial Development Zone, Mulan Town, Xindu District of Chengdu. Sodium oxalate (Na_2_C_2_O_4_), commercial Titania (P25) and L- cysteine were bought from the Sinopharm Chemical Reagent Co, Ltd, and p-benzoquin-one (PBQ) was obtained from the Chengdu Kelong Chemical Reagent Factory.

#### Synthesis of flowerlike BiVO_4_

To synthesize flowerlike BiVO_4_, 0.072 g L-cysteine was dissolved in 4 ml of 4 mol/l HNO_3_ and the mixture was stirred for 30 min, then 2 mmol of Bi (NO_3_)_3_·5H_2_O was dissolved in the mixture and was stirred for 30 min, resulting in solution A. Next, 2 mmol NH_4_VO_3_ was dissolved in 4 ml of 2 mol·L^−1^ NaOH, resulting in solution B. Solutions A and B were mixed together and 50 ml distilled water was added in it, while regulating the pH (2.5), and the mixture was ultrasonically dispersed for 30 min. The mixture was then transferred to a reaction vessel and allowed to react at 180°C for 16 h. Finally, the system was naturally cooled to room temperature and washed with absolute ethanol followed by distilled water about six times. The final product was dried under a vacuum overnight at 60°C for 6 h.

#### Characterization

The crystalline structures of all samples were characterized by X-ray diffraction (XRD) using a Rigaku D/Max-rB diffractometer with Cu Ka radiation. Scanning electron microscopy (SEM) images were acquired with a Zeiss AURIGA field emission microscope (electron high tension (EHT) = 5 kV, work distance (WD) = 8.8 nm; Zeiss, Oberkochen, Germany). The surface chemical environment was analyzed by XPS on a PHI5000 VersaProbe system with monochromatic Al Kα X-rays. Energy dispersive X-ray (EDX) images were acquired with an EDX-100A-4. UV-Vis DRS was performed with a Hitachi U-3010 UV-Vis spectrometer. ESR analyses were performed using a JES FA200 X-band ESR spectrometer operating in the X-band at 0.907 GHz and 0.998 mW.

### Photocatalytic degradation experiment

The photocatalytic activity of BiVO_4_ was assessed by evaluating the degree of photodegradation of Rhodamine B (RhB) solution under visible light at room temperature. In each experiment, 0.10 g of catalyst was added to an aqueous RhB solution (300 ml, 5 mg·L^−1^), and the mixture was magnetically stirred for 30 min in the dark to achieve high dispersion and adsorption–desorption equilibrium between the dye and catalyst. Then, the solution was placed in a 500 mL beaker and positioned 350 mm away from the visible light source. Samples were collected after every 2 h of irradiation, and then centrifuged at 12000 rpm to remove the catalyst powder. The concentration of the remaining dye was monitored by measuring the absorbance of the solution at 552 nm. For comparison, control experiments were performed using P25 as a catalyst under the same conditions. At the same time, we did the repeated test.

### Mechanisms for ·${\text{O}}_{2}^{-}$ and ·OH production

#### ESR experiment

The ESR experiment was primarily focused on the analysis of ·OH and ·${\text{O}}_{2}^{-}$. In the case of ·OH, the whole test was performed in aqueous solution. First, 5 mg of BiVO_4_ was weighed and dispersed in 5 mL water via ultrasound for 20 min; then, argon was bubbled through the solution for 20 min to strip oxygen from the solution; DMPO (100 mM) solution (also bubbled with argon to remove oxygen) was used for hybrid acquisition under visible light. Simultaneously, the same experiment was performed in the presence of oxygen as a control.

There were some differences in the ${\text{O}}_{2}^{-}$ experiment. First, 5 mg of BiVO_4_ was weighed and dispersed in methanol along with a capturing agent, DMPO (100 mM). The solution was then deoxygenated with argon as before, and the test was conducted under visible light. Again, the same experiment was conducted under aerobic conditions as a control. All tests were conducted under neutral conditions (pH = 7).

We also analyzed the DMPO spin-trapping ESR spectra of BiVO_4_ in an aqueous dispersion for DMPO–·OH (1:2:2:1) (Zhou et al., 2012) and in a methanol dispersion for DMPO–·${\text{O}}_{2}^{-}$. The characteristic peaks of DMPO–·OH and DMPO–·${\text{O}}_{2}^{-}$ respectively indicated the production of ·OH and ·${\text{O}}_{2}^{-}$ species in the samples.

To identify whether h^+^ can generate ·OH, we conducted the above ·OH experiment under varying oxygen levels, and then under varying pH (pH = 5, 9, 7, 11).

#### Trapping experiment

The method of trapping experiment is the same to photocatalytic degradation experiment, what different is the addition of scavengers. In these experiments, we used PBQ (0.1 mmol), IPA (0.1 mol·L^−1^), AgNO_3_ (0.1 mmol), and Na_2_C_2_O_4_ (0.1 mmol) as ·${\text{O}}_{2}^{-}$, ·OH, electron, and h^+^ scavengers, respectively (Chen et al., 2013). In each experiment, 0.10 g of catalyst was added to an aqueous RhB solution (300 ml, 5 mg·L^−1^), and the mixture was magnetically stirred for 30 min in the dark to achieve high dispersion and adsorption–desorption equilibrium between the dye and catalyst. Then, the solution was placed in a 500 mL beaker and positioned 350 mm away from the visible light source. Samples were collected after every 2 h of irradiation, and then centrifuged at 12,000 rpm to remove the catalyst powder. The concentration of the remaining dye was monitored by measuring the absorbance of the solution at 552 nm. For comparison, control experiments were performed using BiVO_4_ as a catalyst, or without any catalyst, under the same conditions.

## Results and discussion

### Sample composition and morphology

**Figure 1:**
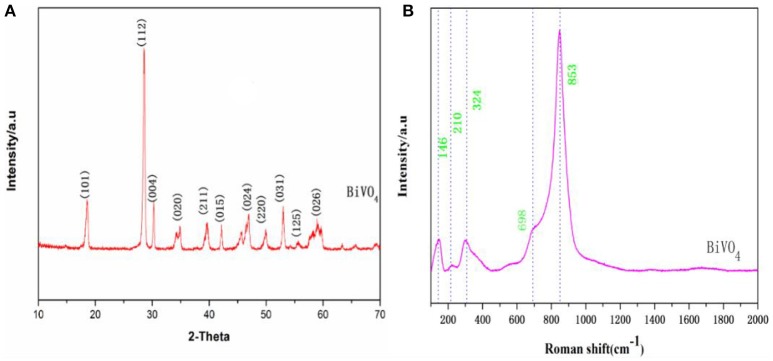
**(A)** XRD patterns of BiVO_4_ **(B)** Raman spectra of BiVO_4_.

X-ray diffraction enables the qualitative analysis and phase identification of a compound. Additionally this analytical technique facilitates determination of crystalline structures (regardless of symmetry measurements, etc.). From the XRD spectrum, the crystalline structure, lattice parameters, and defects (dislocations, etc.) can be determined (Zhang et al., 2006). As seen in Figure 1A, the diffraction peaks of the sample agree well with those for the standard monoclinic BiVO_4_ (JCPDS No. 83–1697), indicating that the material consists of a single phase. Other authors have obtained similar XRD patterns of BiVO_4_ (Zhang et al., 2010; Jiang et al., 2012), suggesting that highly monoclinic scheelite BiVO_4_ was obtained.

Raman spectroscopy is commonly used to determine the structure of prepared BiVO_4_ together with XRD, and we further examined the prepared BiVO_4_ structure with this technique. Based on band component analysis of the Raman and IR spectra (Hardcastle et al., 1991; Gotić et al., 2005; Zhang and Zhang, 2009), we identified Raman bands at 146, 210, 324, 698, and 853 cm^−1^ (Figure 1B). The Raman bands of the prepared BiVO_4_ are quite distinctive and sharp. The band with the highest intensity (853 cm^−1^) was assigned to ν_s_ (V–O) (Zhang and Zhang, 2009), and the weak shoulder at 698 cm^−1^ was assigned to ν_as_ (V–O) (Ye et al., 2012). Medium intensity bands were observed at 324 and 146 cm^−1^. External vibrational modes (rotation/translation) are visible at 210 cm^−1^ (Jiang et al., 2012). These values correspond to the typical vibrations of monoclinic BiVO_4_, although there are minor differences in some parts of the spectrum due to the fact that Raman band positions are sensitive to short-range order, whereas band widths are more sensitive to the degree of crystallinity, defects, disorders, particle size, and/or aggregation of particles (Gotić et al., 2005).

**Figure 2:**
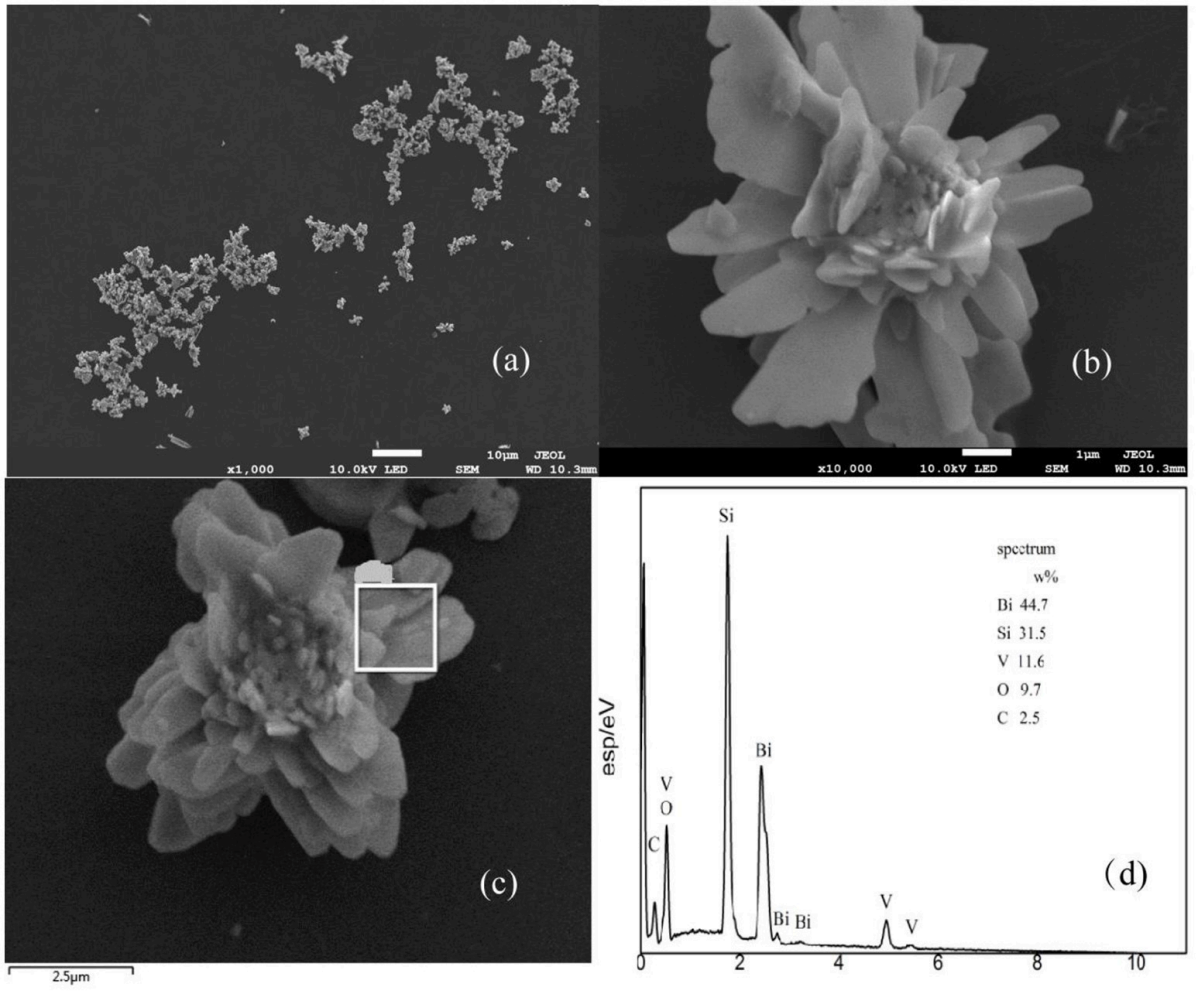
SEM of BiVO_4_ **(a,b)**, and EDX spectra with the corresponding EDX elemental mapping results **(c,d)**.

**Figure 3:**
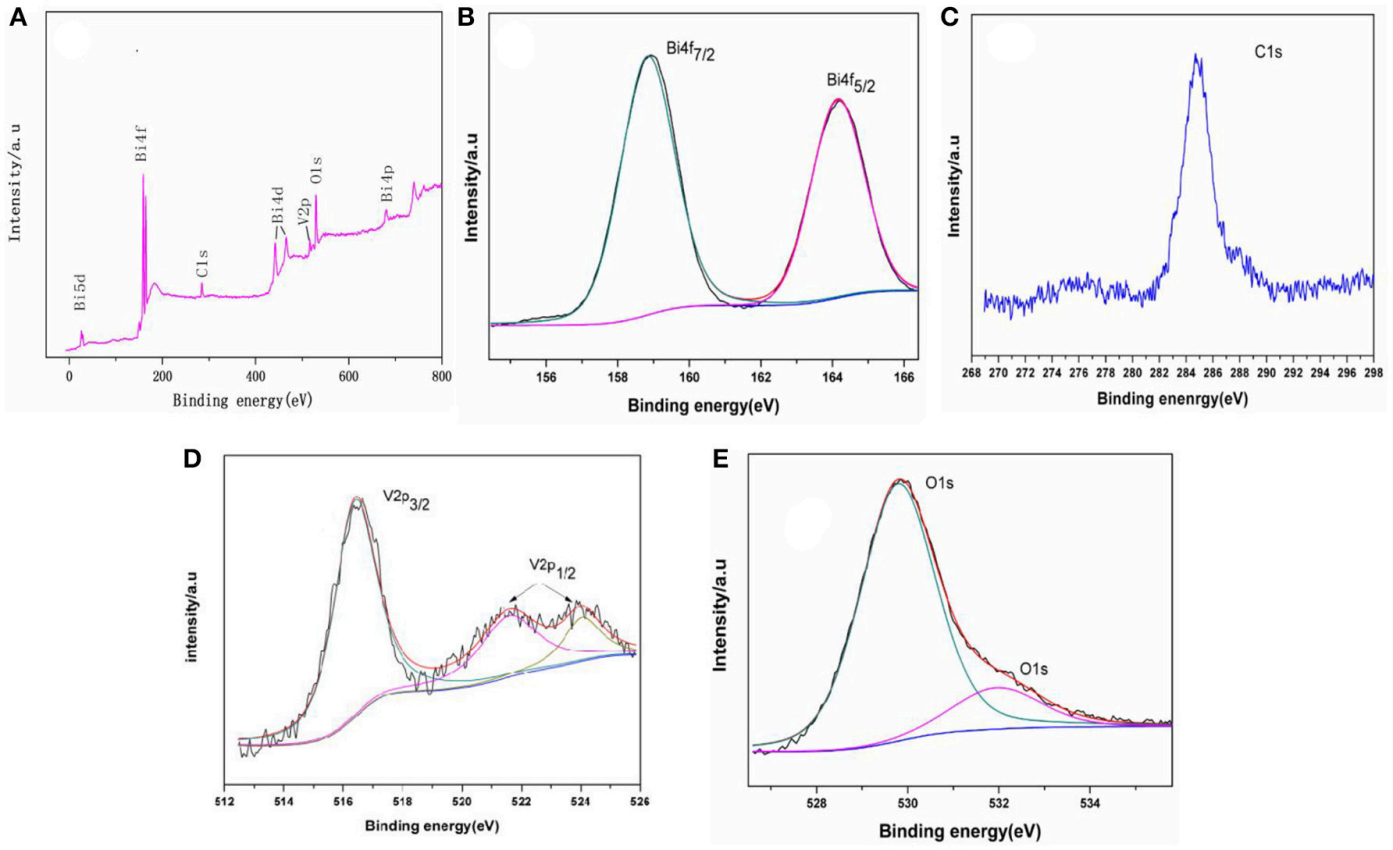
XPS of the samples: **(A)** survey spectra, **(B)** Bi4f5/2 and Bi4f7/2 peaks **(C)** C1s peaks, **(D)** V2p3/2 and V2p1/2, and **(E)** O1s peaks.

The sizes and morphologies of the prepared samples were examined by SEM (Figures 2a,b). The shape of the material is similar to that of the flower, and each of the slices about the sample was about 2–5 μm. Based on the EDX results shown in Figures 2c,d, it can be concluded that the components of the material mainly include V, Bi, O, and Si (Si signals come from the conductive adhesives used in the EDX measurement). These results show the exact composition of the material and are in good agreement with the XPS results (Figure 3).

XPS was used to examine the chemical states of elements in the photocatalytic material. Figure 3A shows the full spectra of BiVO_4_ in the range of 0–800 eV. The survey spectrum shows that the composite contains Bi, O, V, and C. As is reported in some papers (Long et al., 2006; Xu et al., 2016), the form of the Bi is Bi^3+^ in the compound BiVO_4_ and the binding energies for Bi 4f7/2 and Bi4f5/2 are 158.8 and 163.4 eV, respectively (Figure 3B). Figure 3C shows the binding energy of C. The peaks at binding energies of 516.4 eV (V2p3/2), 522.0 eV (V2p1/2), and 524.2 eV (V2p1/2) are visible in Figure 3D. The peaks at binding energying of 516.4 eV (V2p3/2) and 524.2 eV (V2p1/2) indicate the V species in the composite is V^5+^ (Long et al., 2006; Xu et al., 2016; Zhao et al., 2016). And the peak at binding energying of 522.0 eV also represent the element of V (V2p1/2), which means there are some impurities when preparation (Zhao et al., 2016). The three peaks (Figure 3D) all represent V2p, and you can find the same peaks of V in other paper (Jia et al., 2012; Ota et al., 2014). Finally, Figure 3E shows the binding energy of O. Together, these data indicate that the tested sample consists of BiVO_4_ microspheres as the same as other paper although there contains some impurities when preparation (Zhou et al., 2011; Xu et al., 2016).

### Optical properties and degradation properties

**Figure 4:**
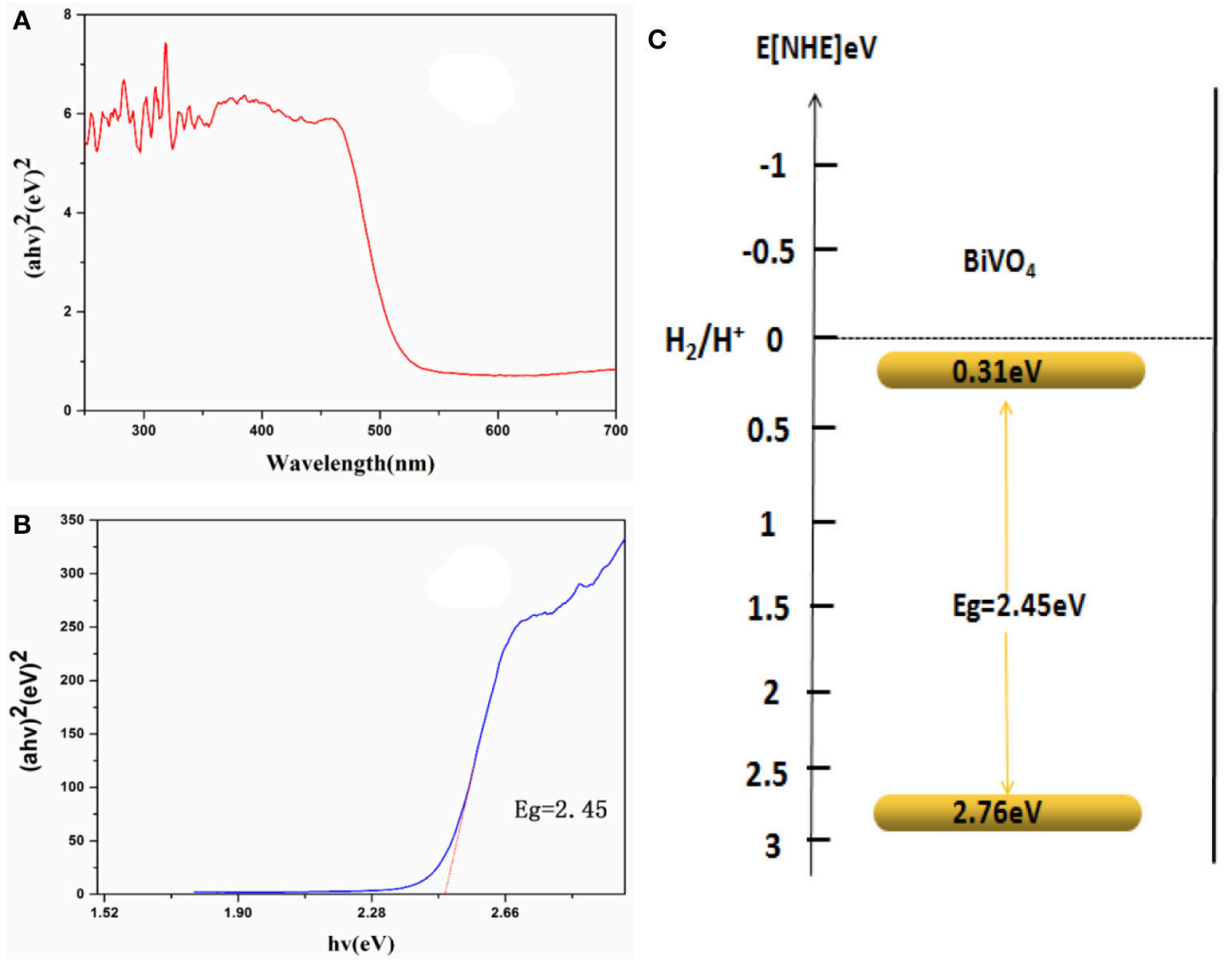
**(A)** UV-Vis DRS spectra. **(B)** Relationship between (αhν)^2^ and the photon energy (hν) of the as-synthesized BiVO_4_ **(C)** Schematic illustration of the band gap structure of BiVO_4_.

Optical absorption properties are key characteristics for a catalyst's photocatalytic activity (Hardcastle et al., 1991). The absorption of visible light by a photocatalyst is mainly due to band-gap transition. To investigate photocatalytic properties, the range of the photocatalyst's absorption wavelength within that of natural light must first be determined. Figure 4A shows the UV-Visible absorption spectra of the obtained BiVO_4_ product, which exhibits strong absorption in both the visible and UV range, indicating its potential as a good photocatalyst for sunlight-driven applications.

As is known to us, energy band structures of a semiconductor continue to be important in determining its photocatalytic activity. And the relationship of absorbance and incident photon energy can be described by the following equation (Cheng et al., 2012):

$$\alpha \text{h}\nu =[\text{A}{\left(\text{h}\nu -\text{Eg}\right)}^{\frac{1}{2}}$$

Where α, ν, and A are the absorption coefficient, light frequency, and proportionality constant, respectively. The band-gap energy (there means direct band-gap), E_g_, of the obtained photocatalyst can be estimated from a plot depicting (αhν)^2^ vs. hν (Cheng et al., 2012; Xu et al., 2016; Zhao et al., 2016). According to the data and corresponding theoretical calculations, the band-gap energies of BiVO_4_ were estimated to be about 2.45 eV as shown in Figure 4B. For a semiconductor, the CBM and VBM can be calculated according to the following empirical equation (Ye et al., 2012): E_CBM_ = χ – Ee −0.5E_g_, where E_CBM_ is the CBM edge potential; χ is the electronegativity of the semiconductor (6.04 for BiVO_4_) (Long et al., 2006); Ee is the energy of free electrons (about 4.5 eV); and E_g_ is the band-gap energy of the semiconductor (2.45 eV, as estimated previously). According to the equation E_VBM_ = E_CBM_ + Eg, the value of E_CBM_ is 0.31 eV and E_VBM_ is 2.76 eV. Similar results were also reported by Long et al (Long et al., 2006). What's more, the value of E_CBM_ and E_VBM_ are different owning to different test or different calculation method in different papers although the band-gap energy is very similar (Zhang et al., 1998; Long et al., 2006). A schematic illustration of the band-gap structure of BiVO_4_ is shown in Figure 4C.

**Figure 5:**
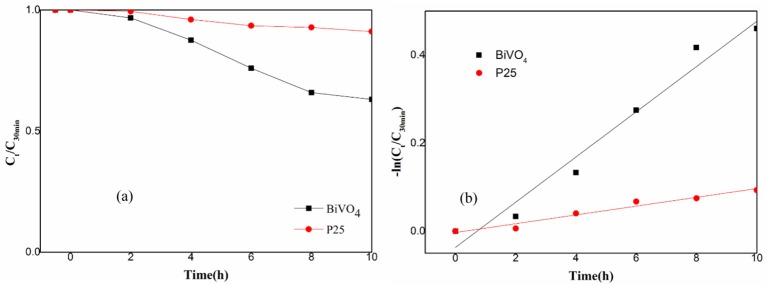
**(a)** Photocatalytic degradation of RhB over BiVO_4_ and P25; **(b)** First order kinetic fitting of Photocatalytic degradation of RhB over BiVO_4_ and P25.

This paper also tested the photocatalytic efficiency of P25 and BiVO_4_ and the result was shown in Figure 5. The Figure 5A experimental results showed that the efficiency of P25 was 9%, while the efficiency of BiVO_4_ was 37%. The Figure 5B was the first order kinetic fitting of the degradation result, both of these results indicated a better efficiency of RhB degradation in the presence of BiVO_4_ compared to the P25, and this exactly represented a good degradation property of BiVO_4_.

### Mechanisms for ·${\text{O}}_{2}^{-}$ and ·OH production

#### ESR analysis

**Figure 6:**
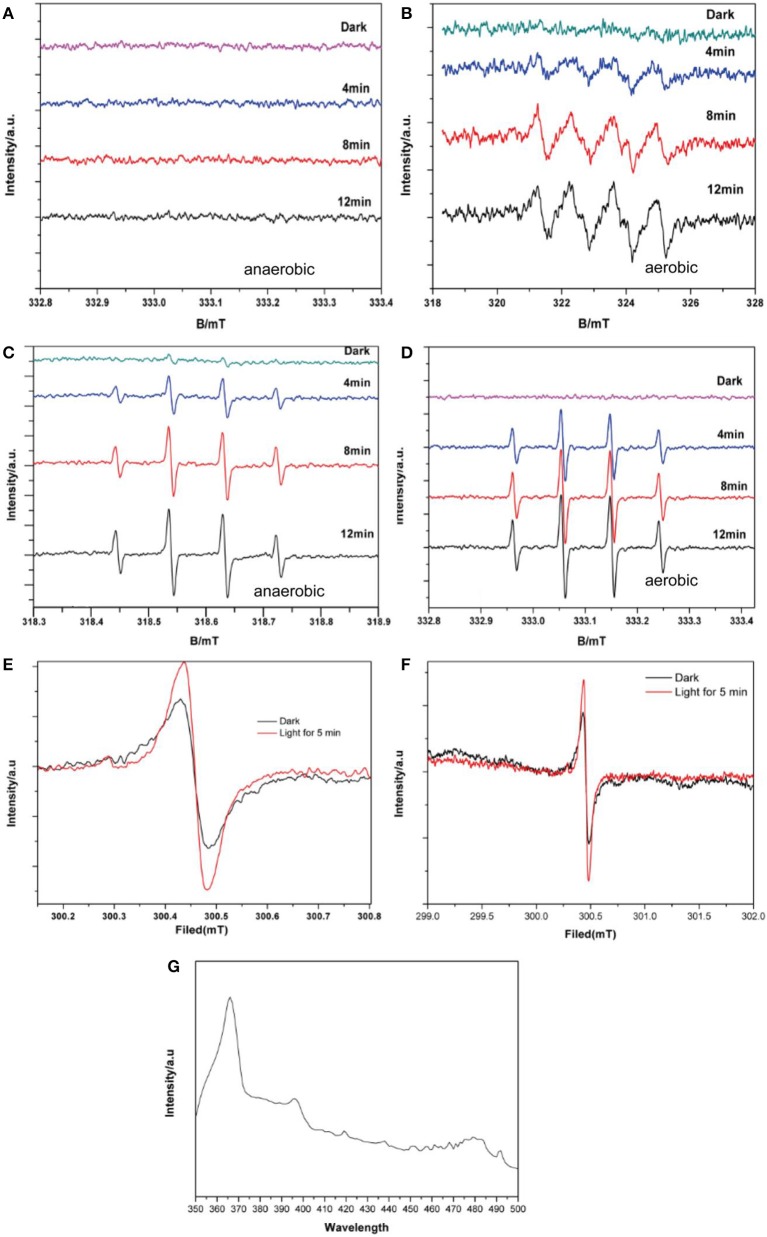
DMPO spin-trapping ESR spectra under visible light for **(A)** DMPO–·${\text{O}}_{2}^{-}$ with BiVO_4_, without O_2_ **(B)** DMPO–·${\text{O}}_{2}^{-}$ with BiVO_4_, with O_2_ **(C)** DMPO–·OH with BiVO_4_, without O_2_ **(D)** DMPO–·OH with BiVO_4_, with O_2_ **(E)** ESR spectra of BiVO_4_ recorded in the dark and under illumination for 5 min **(F)** magnified ESR spectra of BiVO_4_ **(G)** PL spectra of BiVO_4_.

Based on the ESR results as shown in Figures 6A,B, signals with intensities corresponding to the characteristic peaks of DMPO–·${\text{O}}_{2}^{-}$ adducts were observed when the reaction was performed under visible light irradiation, but not when the reaction was performed in the dark. The peak intensities further increased with increased irradiation time. Furthermore, DMPO–·${\text{O}}_{2}^{-}$ signals were observed under aerobic conditions (Figure 6B) but not under anaerobic conditions (Figure 6A). This is consistent with the production mechanism of ·${\text{O}}_{2}^{-}$ via O_2_, as previously reported (Ge et al., 2012b; Ye et al., 2012). Although there still were some studies mentioned no ·${\text{O}}_{2}^{-}$ contained in the degradation system of BiVO_4_ (Ge et al., 2012a; Lopes et al., 2015). As shown in Figures 6C,D, DMPO–·OH was observed under both aerobic and anaerobic conditions, respectively, and the signal intensity was stronger in the aerobic case. These results demonstrate the validity of both aerobic and anaerobic pathways for the production of ·OH. In the first pathway, oxygen, which is an effective electron acceptor, absorbed on the catalyst surface is the main capturing agent targeting photogenerated electrons and can oxidize the hydroxylated products to produce ·OH. The h^+^ produced by the photocatalyst can then oxidize OH^−^ in water to produce ·OH, representing the second possible path. However there is no report for the identification of this phenomenon. The experimental results verified the validity of first path, and to ensure the possibility of the second path, we performed an experiment in which the pH was varied.

The ESR spectrum of BiVO_4_ is shown in Figures 6E,F reveals a magnified section of the same spectrum to provide more detail. These confirm the separation of electron–hole pairs in the BiVO_4_ and provide evidence for the existence of ·OH (Yang et al., 2015). The signal intensity of the 300.5 mT peak (*g* = 2.002) is evidently strong (Figure 6E) in comparison with the peak for the same sample in darkness, indicating the presence of h^+^. In order to further for the explanation about the recombination of electron–hole pairs, we did the photoluminescence (PL) test. PL spectra can reveal the movement, transfer and recombination of photoelectron-hole pairs. It's an effective means to characterize the separation efficiency of semiconductors. In general, the lower the intensity of the emission peak in the PL spectrum, the higher the efficiency of the electron and hole separation in the semiconductor and the higher the photocatalytic activity of the photocatalyst (Gao et al., 2014). From the PL result shown in Figure 6G shows the PL spectra of BiVO_4_ under the excitation wavelength of 325 nm, we can find the strong peak under 367 nm, which means the existence about recombination of electron–hole pairs (Zhao et al., 2017).

**Figure 7:**
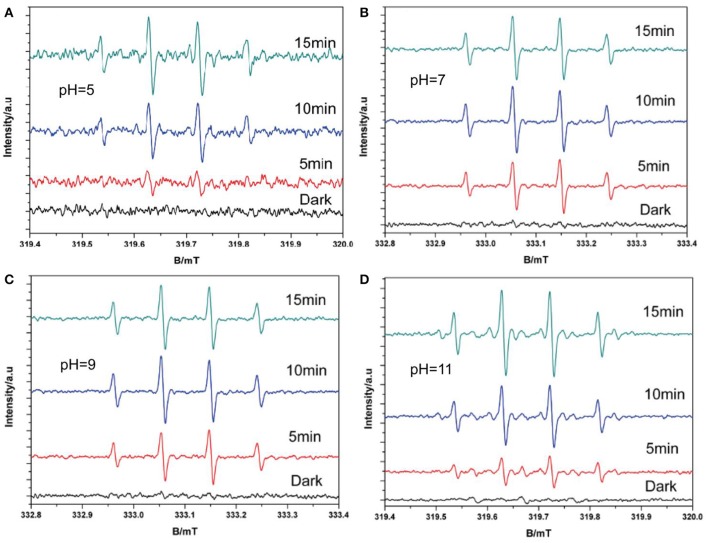
DMPO–·OH with O_2_ spin-trapping ESR spectra under visible light in and varying pH (**A** pH = 5; **B** pH = 7; **C** pH = 9; **D** pH = 11).

According to the results of the pH experiment (Figure 7), the DMPO–·OH signal increased with increasing pH between 5 and 9, which could be found from the signal intensity under 5 min light; however, no obvious increase was observed when the pH increased to 11. This is because more ·OH radicals are produced under alkaline conditions than under acidic or neutral conditions. However, increased alkalinity may also alter the charge of the catalyst surface, thus affecting the production of hydroxyl radicals, which explains the lack of an increase at pH = 11 (Wang et al., 2011). These results confirm the validity of the second path, indicating that ·OH can be produced by the oxidation of h^+^ for OH^−^. Together, the ESR results explain the complete production of ·OH. The trapping experiment was performed to determine the main pathway for ·OH generation.

#### Scavengers study

**Figure 8:**
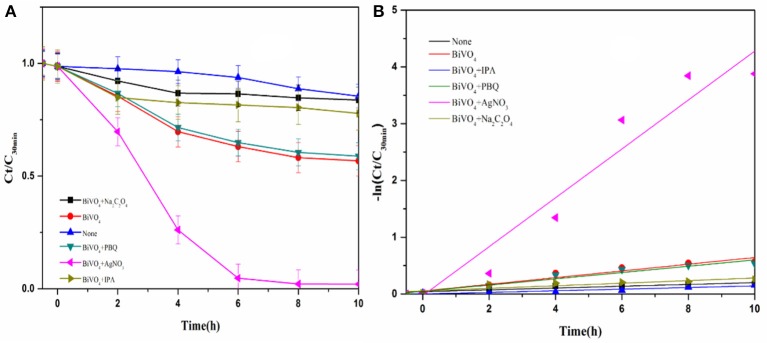
**(A)** Photocatalytic degradation of RhB over BiVO_4_ in the presence of scavengers; **(B)** First order kinetic fitting of Photocatalytic degradation of RhB over BiVO_4_.

The photocatalytic activities of the samples were evaluated by analyzing the degradation of RhB under visible light irradiation. The effect of BiVO_4_ on RhB degradation was obvious when compared to the result without any catalyst (Figure 8). The experimental results indicate complete inhibition of RhB degradation in the presence of IPA, suggesting that ·OH is the main reactive species contributing to the photocatalytic degradation of RhB over BiVO_4_. ·${\text{O}}_{2}^{-}$ also influenced the photocatalytic degradation of RhB, but the effect was less obvious than that of ·OH. As is known to us, the h^+^ and e^−^ are the original active species in the degradation system. The presence of AgNO_3_ captured e^−^ and reduced the recombination of h^+^ and e^−^, as a result it increased the effect of h^+^. At the same time, the h^+^ could further generate ·OH, which promoted the degradation of RhB. Because the ·OH was also the production of hole, we concluded that h^+^ also plays an important role, and similar results were also reported by Zhou et al (Xu et al., 2017). What's more, we found obviously decrease on the addition of Na_2_C_2_O_4_. The capture of h^+^ meant there was no any hole and·OH in the degradation system, thus the degradation effect obviously decreased. As a result, we concluded the importance of hole. And its importance was further confirmed by the slight enhancement of RhB degradation after the addition of Na_2_C_2_O_4_ to capture the photogenerated hole. Furthermore, the addition of AgNO_3_ enhanced the degradation effect, which also demonstrates the strong influence of electron–hole recombination on the degradation process. These results demonstrate that h^+^ can directly degrade RhB, and therefore it plays a significant role in the system, as indicted by the second pathway. In addition, ·OH is mainly generated by h^+^.

#### Elucidate the mechanism

**Figure 9:**
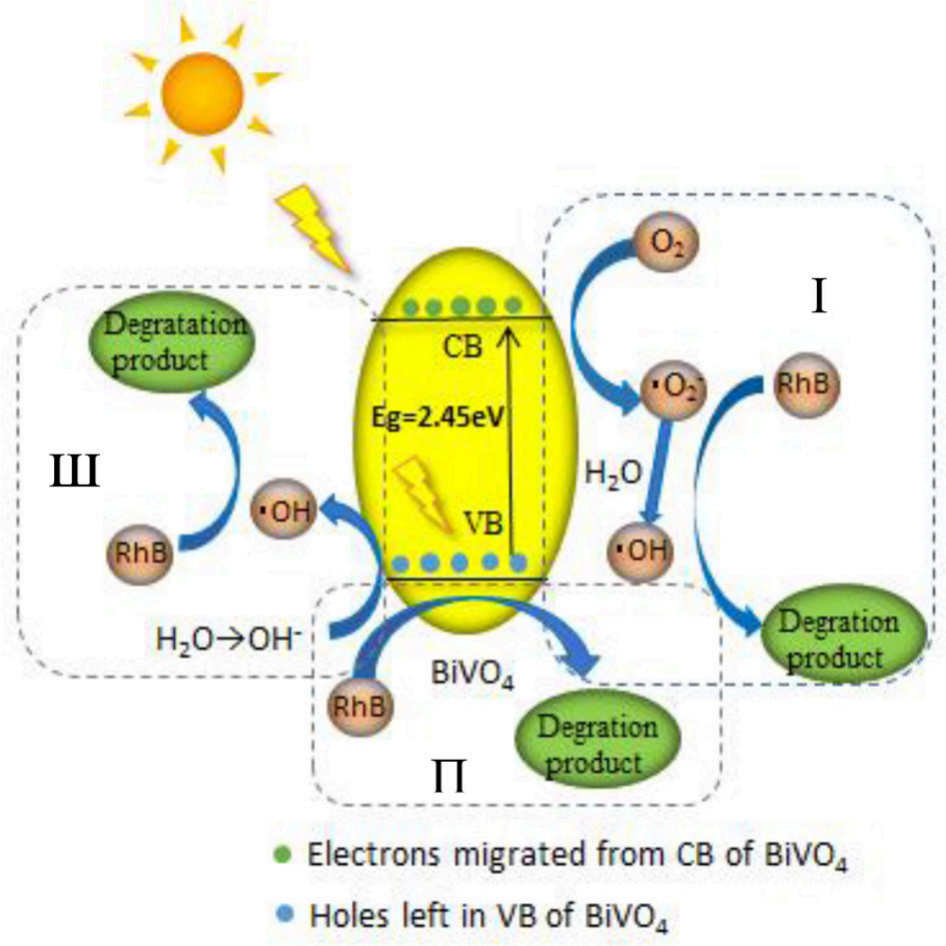
Mechanism of RhB degradation via photocatalysis with BiVO_4_.

We proposed a reaction mechanism based on the results of the experiments described above (Figure 9). First, the adsorbed RhB is photo-activated by visible light, so that electrons can transfer from the singlet excited RhB (RhB^*^) to the CB of BiVO_4_, leaving h^+^ in the VB. Then, the electrons in the CB react with O_2_, resulting in the production of ·${\text{O}}_{2}^{-}$ and ·OH radicals, as illustrated in process I (Figure 9). However, this degradation process is our speculation based on references and the ESR experiment. We cannot ignore the indirect effect of the ·${\text{O}}_{2}^{-}$ on the degradation of RhB owning to its little effect on the production of ·OH based on the trapping experiment result, and this process is not the main degradation mechanism. The ${\text{O}}_{2}^{-}$ is perhaps only participating in a side reaction or maybe can further generate ·OH for the degradation. And the evidence for this process still requires further study. At the same time, h^+^ in VB can oxidize the OH^−^ produced by ionization of water to ·OH, which is the main active free radical for RhB degradation, as shown in process III (Figure 9). Finally, the ·${\text{O}}_{2}^{-}$ and ·OH degrade RhB^+^ and RhB, indicating the major role of ·OH in the degradation system. At the same time, the h^+^ can also degrade RhB^+^ and RhB directly (which can be concluded from the trapping experiment result), as demonstrated in process II (Figure 9). However, the proportion of RhB degraded by this process is minor. So we conclude that there are three processes in the degradation system, the main mechanism is the process III, the process II occur very minor and the process I is the speculation based on our experiment result. Further study would be done for the process I. The entire sequence is summarized in the following reactions:

(R1) $$\begin{array}{rcl} \text{BiV}{\text{O}}_{4}+\text{h}\nu & \to & {\text{h}}^{+}+{\text{e}}^{-} \end{array}$$

(R2) $$\begin{array}{rcl} {\text{O}}_{2}+{\text{e}}^{-}+{\text{H}}^{+} & \to & {\text{H}}_{2}{\text{O}}_{2} \end{array}$$

(R3) $${\text{H}}_{2}{\text{O}}_{2}+{\text{e}}^{-}\to \cdot {\text{O}}_{2}^{-}+{\text{OH}}^{-}$$

(R4) $$\cdot {\text{O}}_{2}^{-}+{\text{H}}_{2}\text{O}\to \cdot \text{OH}+{\text{OH}}^{-}$$

(R5) $$\begin{array}{rcl} h^{+}+\text{O}{\text{H}}^{-} & \to & \cdot \text{OH} \end{array}$$

(R6) $$\begin{array}{rcl} {\text{h}}^{+}+\cdot {\text{O}}_{2}^{-}+\cdot \text{OH}+\text{RhB} & \to & \text{Degration product} \end{array}$$

The mechanism and reactions for flowerlike BiVO_4_ photocatalysis described in this study are the same as those from described in previous research (Liu et al., 2006; Li, 2012; Wei et al., 2013), which are generally accepted as true (Han et al., 2003; Cruz and Pérez, 2010; Tian et al., 2014; Aguilera-Ruiz et al., 2015; Gao et al., 2015). The difference between this study and others is that ESR identifies the production of ·${\text{O}}_{2}^{-}$ and provides a direct analysis of the pathways for the production of ·OH radicals, as shown in R4 and R5. These results indicate that h^+^ can also produce ·OH, a result which has not been identified in previous research (Lam et al., 2014); thus, h^+^ plays an important role in the degradation system by mainly producing ·OH. This result makes it a little more clearly about the production of ·OH in the complete photocatalytic system, of which is one of the most effective active free radicals.

## Conclusion

In summary, we successfully prepared flowerlike BiVO_4_ and characterized the resulting samples using a variety of analytical methods to thoroughly investigate their morphologies and compositions. After the well synthesis of material, we compared the photocatalytic activity of BiVO_4_ to P25 and the result shown a well degradation property of BiVO_4_. Based on the ESR experiment, we found the main free radicals in the reaction system and concluded the production of them by changing test conditions and trapping experiment. The mechanism for RhB degradation via BiVO_4_ photocatalysis was successfully elucidated by these experiments mentioned earlier. The main active free radicals in the reaction system are h^+^ and ·OH. For the production of ·OH, in addition to production by ·${\text{O}}_{2}^{-}$, it can also be produced by oxidation of h^+^ to OH^−^, which is the main pathway for ·OH generation. However, we cannot ignore the indirect effect of the ·${\text{O}}_{2}^{-}$ on the degradation of RhB owning to its little effect on the production of ·OH based on the trapping experiment result. Weather the ·${\text{O}}_{2}^{-}$ can further generate OH still require further future study. More ·OH will be generated if there is more OH^−^ in the degradation system. At the same time, future research should further elucidate the degradation system by conducting experiments under varying pH. This new insight into radical production can be used in tandem with further experimental investigation to help improve photocatalyst performance.

## Author contributions

XX, YS, and ZF: conceived of the study and designed the experiments; YS, SZ, and SX: performed the experiments; YS, DZ, and BZ: analyzed the data; GL: contributed reagents, materials, and analysis tools; YS wrote the paper.

### Conflict of interest statement

The authors declare that the research was conducted in the absence of any commercial or financial relationships that could be construed as a potential conflict of interest. The handling Editor declared a shared affiliation, though no other collaboration, with one of the authors, FZ.
